# Sex differences in hedonic feeding and characterizing the effects of antibiotic-induced microbiome disruption

**DOI:** 10.21203/rs.3.rs-9557496/v1

**Published:** 2026-05-11

**Authors:** Christopher J. Petty, Mindy Isaman, Laura Pallas Perez, Sophie Millard, Max Ortiz, Linnea R. Freeman

**Affiliations:** University of Georgia; Furman University; Furman University; Clemson University; Clemson University; Furman University

**Keywords:** gut microbiome, hedonic feeding, sex differences, antibiotics, behavioral economics, short chain fatty acids, bile acids

## Abstract

**Background:**

Obesity continues to be a public health issue in our country. Additionally, there continues to be a higher incidence of severe obesity for women compared to men. Among the proposed causes of obesity is increased hedonic feeding: food intake driven by pleasure and palatability rather than physiological hunger. While hedonic feeding is not the sole culprit for the obesity epidemic, it is a major contributing factor. Emerging evidence shows that the gut microbiome impacts feeding behavior and studies have shown that individuals with obesity exhibit an altered gut microbiome.

**Methods:**

Here, we used a novel behavioral economics (BE) approach to evaluate hedonic feeding in male and female Sprague-Dawley rats for a high-fat palatable (HFP) reward pellet, before and after antibiotic administration. Specifically, we measured demand elasticity (α), the rate at which demand falls when the price or effort required increases, and demand at null cost (Q_0_), a prediction of consumption at null effort extrapolated from the animals’ consumption at low price.

**Results:**

We determined a higher demand at null cost (Q_0_) for the HFP reward pellet for females compared to males, as we have observed previously. Next, we administered an antibiotic cocktail in the drinking water to disrupt the gut microbiome and investigate a role of the gut microbiome in hedonic feeding. Female rats administered antibiotics continued to have a higher demand at null cost compared to male control rats, but no statistically significant differences were determined between male and female rats administered antibiotics. We characterized the fecal microbiome genus-level composition and short chain fatty acid (SCFA) levels before and after antibiotic administration. We also characterized serum SCFA and bile acid levels at the end of the study.

**Conclusions:**

We did not determine a significant effect of antibiotics on hedonic feeding, despite disruption to the fecal microbiome. Additionally, we did not observe striking baseline sex differences in fecal microbiome diversity and composition. This brings to question whether the gut microbiome contributes to sex differences in hedonic feeding. More research will be necessary for network factors such as microbiome – bile acid effects on feeding that exhibit sex differences.

## Plain English summary

Obesity incidence has continued to rise in the United States as well as the world. The prevalence of obesity affects more women than men, especially severe obesity. Some of the proposed causes of obesity/severe obesity are increased access to calorie-dense foods, decreased energy expenditure, and increased hedonic feeding: food intake driven by pleasure and palatability rather than physiological hunger. While hedonic feeding is not the sole culprit for the obesity epidemic, it is a major contributing factor as the widespread availability of palatable foods can lead to chronic overconsumption. We evaluated hedonic feeding using a behavioral economics approach in male and female rats. We also characterized the fecal microbiome and multiple metabolites related to the gut microbiome. This study aimed to evaluate the role of the gut microbiome in sex differences in hedonic feeding. We determined that female rats consume more of the high fat, high sugar reward compared to males under no-cost conditions of the behavioral economics paradigm. However, we did not determine significant differences in the composition of the gut microbiome at the genus level that could explain the sex differences in hedonic feeding. Lastly, we characterized sex differences in the bacterial composition of the fecal microbiome, fecal and serum short chain fatty acid, and serum bile acid composition following antibiotic treatment. More research will be necessary for network factors such as microbiome – bile acid effects on feeding that exhibit sex differences.

## Background

Obesity (body mass index ≥ 30) and severe obesity (body mass index ≥ 40) prevalence in the United States have increased between 2000 and 2023 from 30.5% to 40.3% and 4.7% to 9.4%, respectively (CDC). Specifically, the prevalence of obesity for adult women in 2000 was 33.4% compared to 27.5% for adult men. In 2023, the prevalence of obesity for adult women was 41.3% compared to 39.2% for adult men. The prevalence of severe obesity for adult women in the United States was 6.2% in 2000 compared to 3.1% for adult men; it was 12.1% for adult women in 2023 compared to 6.7% for adult men [3]. Sex differences in obesity prevalence have decreased as obesity rates have risen, but the sex differences in severe obesity remain. Among the proposed causes of obesity/severe obesity are increased access to calorie-dense foods, decreased energy expenditure, and increased hedonic feeding: food intake driven by pleasure and palatability rather than physiological hunger. While hedonic feeding is not the sole culprit for the obesity epidemic, it is a major contributing factor as the combination of food-driven reward seeking and widespread availability of palatable foods can lead to chronic overconsumption.

Sex differences in palatable food consumption have been determined in preclinical studies. Female rats consume more palatable food and escalate their intake faster compared to males [4–7]. We have previously shown that females have a higher demand at null cost for three different palatable rewards using a behavioral economics (BE) single-session paradigm, a de-escalating fixed ratio operant task designed to evaluate a reward’s value and measure how much effort the subject is willing to expend to earn that reward [8]. These sex differences in hedonic feeding could provide support for observed sex differences in severe obesity.

One factor that impacts feeding behavior is the gut microbiome composition [9–11]. Germ-free mice consume more sucrose compared to controls when administered a high concentration of sucrose solution. Male mice given antibiotics to deplete their gut microbiome have been shown to consume more sucrose pellets compared to vehicle controls. Furthermore, antibiotic-treated mice that received fecal transplants from specific pathogen-free donors reduced sucrose pellet consumption, comparable to vehicle controls [9]. These findings suggest that the microbiome plays a role in reward-driven feeding. Studies have also shown that individuals with obesity exhibit an altered gut microbiome [12]. This difference in the microbiome has been studied in animal models as well, revealing that a fecal microbiota transplant from obese to germ-free mice leads to weight gain in the mice that received the obese microbiota profile [12, 13].

Here, we used a novel, behavioral economics approach to evaluate hedonic feeding in male and female Sprague-Dawley rats for a high-fat palatable (HFP) reward pellet before and after antibiotic administration. This behavior analysis focused on hedonic feeding behavior; animals were not obese. Specifically, we measured demand elasticity (α), the rate at which demand falls when the price or effort required increases, and demand at null cost (Q_0_), a prediction of consumption at null effort extrapolated from the animals’ consumption at low price. We determined a higher demand at null cost for the HFP reward pellet for females compared to males, as we have observed previously [8]. Furthermore, we investigated hedonic feeding behavior before and after antibiotic administration; an antibiotic cocktail was administered in the drinking water for one month in order to disrupt the gut microbiome. We analyzed hedonic feeding behavior, homecage chow consumption, body weights, fecal microbiome composition and diversity, fecal and serum short chain fatty acid composition, and serum bile acid composition. While demand at null cost (Q_0_) values were significantly different between Male Control and Female Control as well as Male Control and Female Antibiotics pre-treatment, we did not determine a statistically significant difference between any of the groups post-treatment. Overall, there was no sex difference in hedonic feeding in post-treatment groups and antibiotics did not have a statistically significant effect on hedonic feeding; however, antibiotics did alter the fecal microbiome and decreased fecal SCFA, serum SCFA, and serum bile acid composition.

## Methods

### Animals

Male and female Sprague Dawley rats (Charles River Laboratories; n = 45) were individually housed and kept on a reverse 12-hour-light schedule, with behavior experiments occurring during the dark cycle. In order to monitor food consumption, water intake, and the fecal microbiome, rats were housed individually, as done previously in other dietary studies [8, 16–19]. Despite single housing, rats were housed in the same room to expose them to olfactory, visual, and auditory stimuli produced by other subjects and were therefore not completely isolated [20]. Rats were frequently handled due to daily training or testing throughout the study. Rats had ad libitum access to chow (PicoLab ^®^ Laboratory Rodent Diet 5L0D, irradiated, Lab Supply, Northlake, TX), consisting of 13% calories from fat, 58% from carbohydrates, and 29% from protein, and water throughout the duration of the experiment outside of the antibiotic phase where water was substituted with an antibiotic cocktail for a subgroup of rats. Body weights, food consumption, and water intake were monitored weekly throughout the study. Body weights were also monitored daily during BE testing phases; we normalize Q_0_ values to body weight so we needed daily measurements for those calculations. All protocols and procedures followed the NIH Guidelines for the Care and Use of Laboratory Animals and were approved by the Furman University Institutional Animal Care and Use Committee.

### Reward Pellet

During BE training and testing, a high-fat palatable (HFP) 45 mg pellet (Bio-Serv, Frenchtown, NJ) was utilized (0.72 kcal/g protein, 2.11 kcal/g fat, 1.77 kcal/g carbohydrate; Product #F06162). Hydrogenated cottonseed oil and soybean oil constitute the fat sources in this food reward. This pellet also contains dextrose and sucrose contributing to the palatability. In addition to the nutrients mentioned above, the pellet contains a standard mineral and vitamin mix.

### Study Design

Rats were trained to press an active lever for the HFP pellet in an operant chamber (Med Associates) housed inside a sound-attenuating cubicle. The cubicle contained a red house light, two retractable levers with white cue lights above them, a food hopper, and a tone generator to reinforce the pressing of the active lever inside the chamber. Rats were trained on fixed ratio 1 (FR1) training followed by FR3, FR10, FR32, and FR100, each for a minimum of 2 days, during which they had to meet criteria for at least 50 lever presses (except FR100, which did not have a minimum lever press criteria and was only administered one day to avoid extinction). Animals not progressing through the fixed ratio training were removed from the study (n = 11 males and n = 2 females). After one day of FR100 training, rats started the BE testing. During the 105-minute BE session, a 5-minute “active period” was signaled by the house light’s illumination and the levers’ extension. During the active periods, responses to the active lever delivered the reward pellets on an FR schedule. The first active period was the highest schedule of reinforcement at FR100, followed by FR32, FR10, FR3, and finally, FR1. There was no maximum for pellets earned during testing. Instead, 20-minute time-out periods signaled darkness in the chamber and retraction of the levers; the time-out and the reverse order of FR schedules were employed to limit satiation. Responses on an inactive lever were not reinforced. This design produced full demand curves. Rats were administered at least 6 BE sessions until α (demand elasticity) varied less than 25% across the last three days. While this novel, behavioral economics approach includes all FR values during a single session, multiple testing sessions allow for assessment of consistent behavior, providing a “stable” α value and the associated Q_0_ value for that session. Once the rats reached a stable baseline α, they were treated with an antibiotic cocktail containing 0.5g/L of vancomycin, 1.0g/L ampicillin, and 1.0g/L neomycin added to the homecage drinking water (Cayman Chemical Company, Ann Harbor, MI). Antibiotic water was monitored and changed twice weekly.

After one month of antibiotic treatment, the rats completed a second round of BE testing, following the abovementioned protocol. The antibiotic water also continued during this second round of BE testing. A control group received normal drinking water for one month and then completed BE testing, still receiving normal drinking water. After completion of the protocol, rats were euthanized and trunk blood was collected.

### Fecal Microbiome Analysis

Fecal samples were collected via sterile technique on days 5 and 6 of the initial BE testing as well as on days 5 and 6 of the second BE testing. Samples were sent to Clemson University Genomics and Bioinformatics Facility (CUGBF) for extraction, library preparation, and sequencing. DNA was extracted from fecal pellets with the DNeasy UltraClean Microbial kit (Qiagen, #1224-50). Library preparation and 16S rRNA gene amplicon sequencing was conducted following the protocol developed by Kozich et al. [21]. Briefly, the V3-V4 region of the 16S rRNA gene was PCR-amplified using barcoded dual-index primers. Upon confirmation of a correctly sized PCR product using gel electrophoresis (Invitrogen, #G401002), PCR products were normalized using the SequelPrep plate kit (Life Technologies, #A10510-01) and pooled per 96-well plate. Each pool was quantified using qPCR (KapaBiosystems, #KK4854) and sized using the Agilent Bioanalyzer high-sensitivity DNA kit (Agilent, #5067 – 4642). Multiplexed pooled amplicon libraries were sequenced paired end 2 x 300 cycles on the Illumina NextSeq2000 platform with a 10% PhiX spike according to manufacturer’s protocol.

Initial amplicon sequence processing was conducted in QIIME2 v.2024.2 [22]. Raw sequences were quality filtered, denoised, and assigned to Amplicon Sequence Variants (ASVs) using the DADA2 plugin [23]. Representative sequences were aligned using a multiple sequence alignment program (MAFFT) and a phylogenetic tree was generated with fasttree [24]. ASVs taxonomy were assigned using the SILVA 138 database. The final ASVs table was rarefied to 25.9k reads per sample and used for subsequent analyses.

### Short Chain Fatty Acid (SCFA) and Bile Acid Analysis

The Duke Proteomics and Metabolomics Core Facility performed SCFA and bile acid analysis. Each fecal sample (50–100 mg) was placed in bead blaster CK-14 homogenization tubes (Bertin Corp) and homogenized using the Precellys 24 bead blaster (Bertin Instruments) at 4°C for three cycles of 10 seconds each at 10,000 rpm, with a 60-second pause between bursts. After homogenization, sample extracts underwent centrifugation at 15,000 relative centrifugal force for 15 minutes at 4°C. Data collection utilized LC–MS/MS on a Waters Xevo TQ-S mass spectrometer. Calibration curves were established for each analyte, and a ^13^C_6_ internal standard was used for compound quantification (via ^13^C_6_ NPH derivatization reagent). Data analysis was performed using Skyline software (www.skyline.ms), with initial concentrations reported in μM. To convert concentrations, the mass of feces homogenized and extracted was included, along with the volume of solvent added, resulting in nmol/mg concentrations.

Serum samples were also analyzed for SCFAs. This analysis was performed with the Sciex QTrap 6500 + system (Framingham, MA) with Waters Acquity I-class plus UPLC. Software Analyst 1.7.3 was used for data acquisition. For sample preparation, 20 μL serum was mixed with 40μL ethanol, and then kept at −20° C for 20 min, followed by vortex and then centrifugation at 15,000 rcf for 4 minutes at 10° C. All data was analyzed in Skyline v23.1.0.455 (www.skyline.ms) which includes raw data import, peak integration, and a quadratic regression fit with 1/x2 weighting for the calibration curves for SCFA. The SCFA method quantified 12 SCFAs commonly found in biological samples: acetic acid, propionic acid, iso-butyric acid, butyric acid, 2-methyl butyric acid, iso-valeric acid, valeric acid, 3-methyl valeric acid, iso-caproic acid, caproic acid, heptanoic acid, and octanoic acid. This validated method is based on previous work published by Han et al. [25].

Serum samples were analyzed with the Biocrates Bile Acids assay (Biocrates, Innsbruck, Austria), which quantifies 20 bile acids. The serum samples were centrifuged at 10,000 rcf for 2 minutes in a refrigerated (4°C) centrifuge then stored on ice until addition to the bile acids kit plate. Samples were prepared in strict accordance with the Biocrates detailed protocol. Addition of 10 μL of the supplied internal standard solution to each well of the 96-well extraction plate was followed by drying under a gentle stream of nitrogen. Study samples, calibration standards, and QCs were added in 10 μL aliquots to the appropriate wells. The plate was then dried a second time under a gentle stream of nitrogen. The samples were eluted with methanol then diluted with water. Sample analysis of bile acids was performed by a Waters ultra-high pressure liquid chromatography (UPLC) tandem mass spectrometric method using a reversed phase analytical column for analyte separation. Selective analyte detection was accomplished by use of a Xevo TQ-S triple quadrupole tandem mass spectrometer operated in multiple Reaction Monitoring (MRM) mode, in which specific precursor to product ion transitions were measured for every analyte and stable isotope labeled internal standard. Pools of the study samples (SPQC) were injected before, during, and after the study samples in order to measure the performance of the assay across the sample cohort. The UPLC-MS/MS data were directly imported into Biocrates WebIDQ^™^ software for peak integration, calibration, and concentration calculations.

### Estrus Cycle Analysis

During BE testing, the estrus cycle was evaluated via vaginal lavage. Female rats were acclimated to the action of a vaginal lavage beginning on the first day of FR32 training through the action of handling and imitation of the lavage with an empty and sterile transfer pipette. Therefore, females were acclimated to vaginal lavage prior to BE testing. When the rats entered the testing phase, researchers collected cell samples via vaginal lavage using a 0.9% NaCl solution until stabilization on the BE task occurred. Collected samples were smeared onto a glass slide, stained with Quik-Dip Hematology Stain (Mercedes Medical, FL) and evaluated under the microscope in order to characterize the cycle phase (estrus, proestrus, diestrus, and metestrus). The cycle phase during the test day that a stable α value was reached is shown in Fig. 2B.

## Statistical Analysis

Statistical tests were completed using GraphPad Prism (GraphPad Software, La Jolla, California, USA, www.graphpad.com) and R v.4.4.1. Body weight gain, food consumption, water consumption, BE values, and fecal SCFAs were analyzed using a linear mixed-effects model with Time (pre vs post), Sex, and Treatment as fixed effects and animal ID as a random effect. Tukey’s multiple comparisons test was conducted to correct for multiple testing. Serum bile acids and SCFAs were analyzed using a one-way ANOVA since serum was only collected at the end of the study; a pre vs post analysis was not conducted.

Microbiome diversity analyses were performed in R v.4.4.1 using phyloseq [26], vegan [27] and microeco packages [28]. In alpha-diversity, the Kruskal–Wallis rank-sum test was subsequently used to calculate the significance of mean differences in variables between treatments, and the pairwise Wilcoxon rank-sum test was used to compare significant differences between groups [29]. P-value correction for multiple testing was performed according to the Benjamini-Hochberg FDR method [30]. Beta diversity was assessed using Principal Coordinates Analysis (PCoA) [31] based on Bray–Curtis and Weighted Unifrac distance matrices. Permutational Multivariate Analyses of Variance (PERMANOVA), with 999 permutations, was used to test for significant differences between groups [29]. Variations within communities were determined by distance-based tests for homogeneity using the betadisper function [32]. Differential abundant taxa were determined using LEfSe (Linear discriminant analysis Effect Size) [33]. Co-occurrence networks at genera level were constructed for water- and antibiotic-treated groups. ASVs with low relative abundance were removed and Spearman correlations were calculated with Benjamini-Hochberg FDR P value correction [30]. Only significant relationships with a correlation coefficient (ρ) ≥ ± 0.6 and P < 0.01 were selected and translated into networks. The networks were further visualized using the interactive platform Gephi v.0.10.1 [34].

## Results

**Figure 1:**
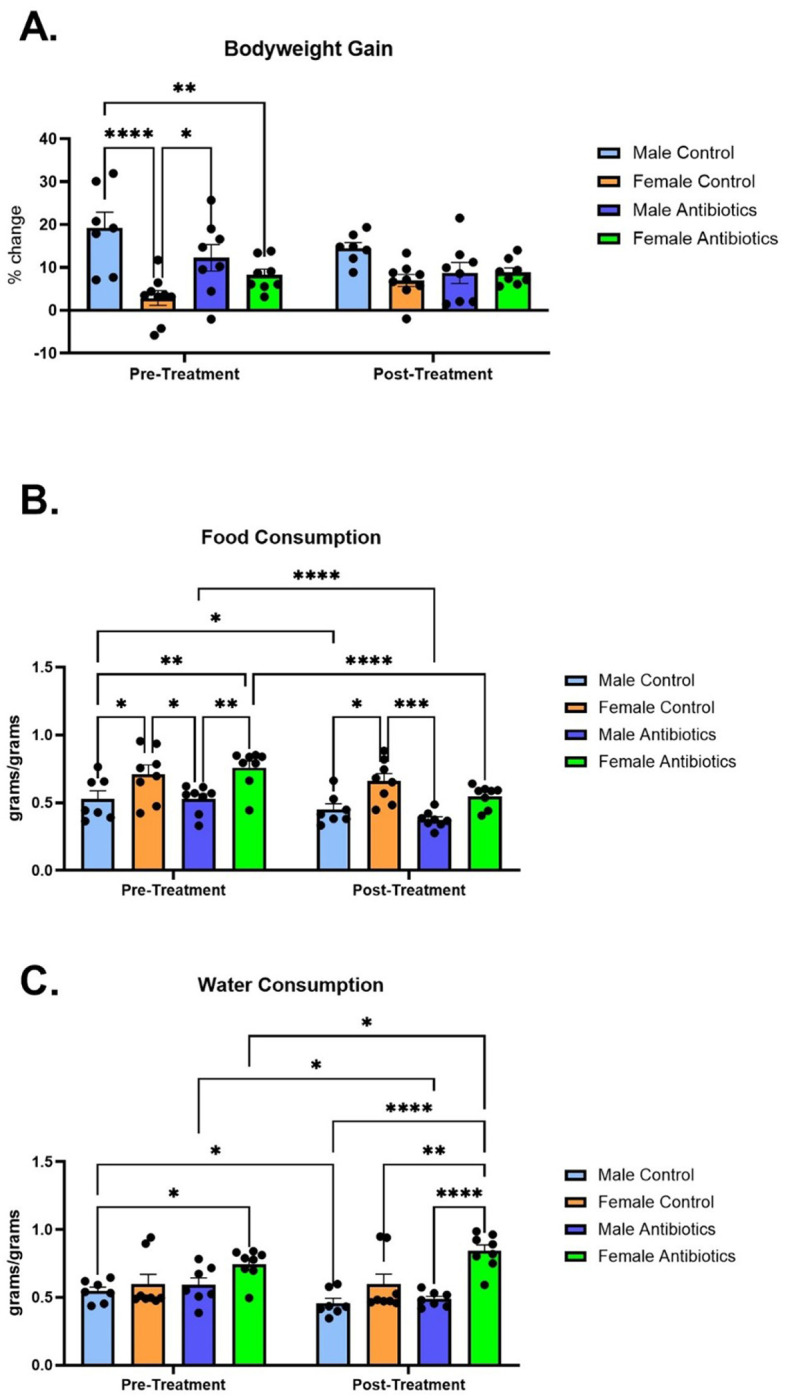
Animal Measurements. Body weights, food consumption, and water intake were monitored weekly during the study. **A)** Pre-treatment, Male Control gained significantly more weight than Female Control (*p* <0.0001) and Female Antibiotics (*p*= 0.0048). Male Antibiotics also gained significantly more weight than Female Control (*p* = 0.0128). There were no statistically significant differences in body weight gain post-treatment. **B)** There was a significant sex x treatment effect (F(3,27) = 6.649, *p* = 0.0017), time effect (F(1,27) = 80.27, *p* < 0.0001), and sex x treatment x time interaction effect (F(3,27) = 7.165, *p* = 0.0011) for food consumption. **C)**There was a significant sex x treatment effect for water consumption (F(3,27) = 7.677, *p* = 0.0007) and a sex x treatment x time interaction effect (F(3,25) = 4.813, *p* = 0.0088).

The percentage of body weight gain (change in weight divided by starting weight), from the start of the experiment to the end of the first session of BE testing (Pre-Treatment), and then the end of the first session of BE testing to the end of the second session of BE testing (Post-Treatment), was compared between four groups: Male Control, Male Antibiotics, Female Control, Female Antibiotics (Figure 1A). A significant sex x treatment effect was determined (F(3,56) = 10.78, *p* <0.0001). Pre-treatment, Male Control gained significantly more weight than Female Control (*p* <0.0001) and Female Antibiotics (*p* = 0.0048). Male Antibiotics also gained significantly more weight than Female Control (*p* = 0.0128). These changes in body weight were prior to antibiotic treatment, or control, water treatment; this confirms no statistically significant differences for Male Control vs. Male Antibiotics and Female Control vs. Female Antibiotics at baseline. There were no statistically significant differences in body weight gain post-treatment. Homecage chow consumption (Figure 1B) and water consumption (Figure 1C) were evaluated weekly throughout the study. Average food consumption and water consumption, pre-treatment and post-treatment, were normalized to the animal’s body weight. There was a significant sex x treatment effect (F(3,27) = 6.649, *p* = 0.0017), time effect (F(1,27) = 80.27, *p* < 0.0001), and sex x treatment x time interaction effect (F(3,27) = 7.165, *p* = 0.0011) for food consumption. Female Control consumed significantly more homecage food (normalized to body weight) compared to Male Control (*p* = 0.0492) and Male Antibiotics (*p* = 0.0389), pre-treatment. Female Antibiotics consumed significantly more homecage food compared to Male Control (*p* = 0.0074) and Male Antibiotics (*p* = 0.0052), pre-treatment. Given that these were baseline, pre-treatment measurements, no statistically significant differences between Male Control and Male Antibiotics or Female Control and Female Antibiotics were observed, as expected. Post-treatment, Female Control continued to consume more homecage food compared to Male Control (*p* = 0.0169) and Male Antibiotics (*p* = 0.0004). Post-treatment, Female Antibiotics consumed less homecage food, but this was not significantly different from Female Control (*p* = 0.3394). There was a significant sex x treatment effect for water consumption (F(3,27) = 7.677, *p* = 0.0007) and a sex x treatment x time interaction effect (F(3,25) = 4.813, *p* = 0.0088). Pre-treatment, Female Antibiotics consumed significantly more water than Male Control (*p* = 0.0411). Post-treatment, Female Antibiotics drank more water compared to all other groups:

Female Antibiotics vs. Male Control: *p*< 0.0001, Female Antibiotics vs. Male Antibiotics: *p*< 0.0001, and Female Antibiotics vs. Female Control: *p* = 0.0052. Given that females drank the most antibiotic water, they did receive a higher dose of antibiotics compared to males (*p*<0.0001).

**Figure 2:**
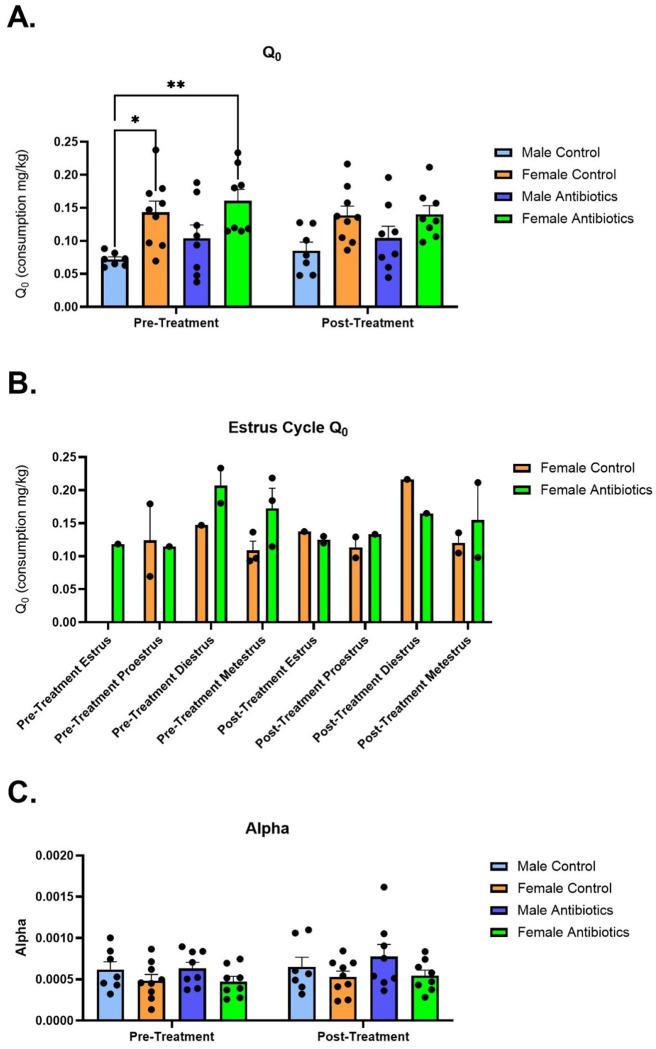
Behavioral Economics and Estrus Cycle Analysis. **A)** A significant sex x treatment effect was determined for Q_0_ values (F(3, 28) = 6.477; *p* = 0.0018). Q_0_ values were significantly higher (*p* = 0.0119) for Female Control compared to Male Control. Female Antibiotics Q_0_ was also significantly higher than Male Control (*p* = 0.0016). There was no statistically significant difference between Male Antibiotics and Female Antibiotics (*p* = 0.0627). **B)** Q_0_ values pre-treatment and post-treatment, with the estrus cycle characterized for that final day of BE testing when the female had a stable α value. There are not enough data points to conduct statistical tests on these data. **C)** No statistically significant sex or treatment differences were determined for α at of the experiment.

A significant sex x treatment effect was determined for Q_0_ values (F(3, 28) = 6.477; *p* = 0.0018). Q_0_ values were significantly higher (*p* = 0.0119) for Female Control compared to Male Control (Figure 2A). Female Antibiotics Q_0_ was also significantly higher than Male Control (*p* = 0.0016). There was no statistically significant difference between Male Antibiotics and Female Antibiotics (*p* = 0.0627). There was also no statistically significant difference for Male Control vs. Male Antibiotics (*p* = 0.5019) or Female Control vs. Female Antibiotics (p = 0.2747). If pre-treatment groups are collapsed and compared by sex: female pre-treatment has significantly higher Q_0_ values compared to male pre-treatment (*p* = 0.0110) as determined by Welch’s t-test. Q_0_ individual values pre- vs. post-treatment are further compared in Figure S1. No statistically significant differences in values pre- vs. post-treatment were determined with a paired t-test within each sex and treatment group. The estrus cycle was characterized after collection of samples via vaginal lavage throughout BE testing. Female rats stabilized on the BE paradigm during the four stages of their cycle: diestrus, proestrus, estrus, and metestrus. Figure 2B displays the Q_0_ values pre-treatment and post-treatment, with the estrus cycle characterized for that final day of BE testing when the female had a stable α value. There are not enough data points to conduct statistical tests on these data. No statistically significant sex or treatment differences were determined for α at any stage of the experiment (Figure 2C). Table 1 displays the average number of active and inactive lever presses as well as pellets earned for males and females during the training period, prior to the BE testing.

**Figure 3:**
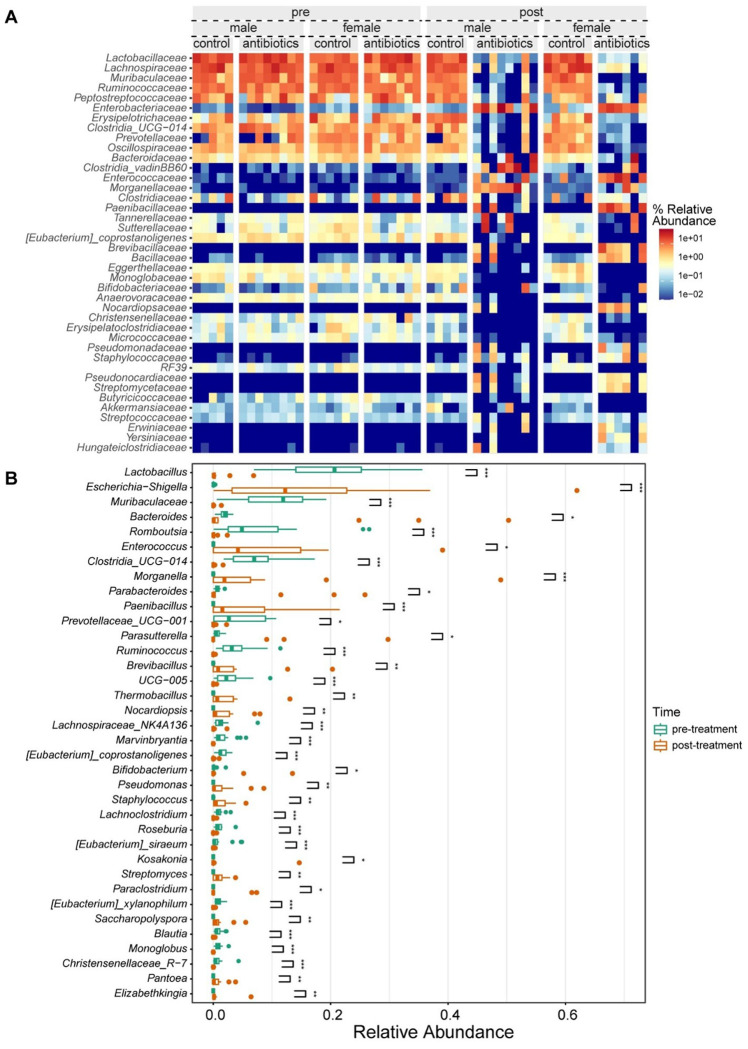
Alteration of the rat fecal microbiome composition following antibiotic treatment. **A)** Log_10_ mean relative abundance of the 40 most abundant bacterial genera across experimental groups. **B)** The 20 genera with the largest differences in relative abundance (LDA > 3.5, *p* < 0.05) between pre- and post-antibiotic treatment, as identified by Linear Discriminant Analysis Effect Size (LEfSe). Significance between pre- and post-antibiotic treatment was determined using the Wilcoxon rank-sum test (*p < 0.01, **p <0.001, ***p < 0.0001).

The composition of the fecal microbiome was altered by antibiotics. Many of the top 40 most abundant genera showed significantly decreased relative abundance following administration of the antibiotic cocktail in both male and female rats (Figure 3A). Post-antibiotics, males had increased relative abundance of *Clostridia vadin BB60* and *Morganellaceae* compared to pre- treatment and water groups. Although not statistically significant, these genera appeared to be higher in abundance in males post-antibiotics compared to females post-antibiotics. Conversely, females showed increased relative abundance of *Bacillaceae, Nocardiopsaceae, Brevibacillaceae, and Paenibacillaceae* compared to pre-treatment and water groups. Once again, no statistical significance in the abundance of these genera was found between sexes after antibiotic treatment although their abundance does appear to be higher in females compared to males post-antibiotics. Linear Discriminant Analysis Effect Size (LEfSe) was used to compare the relative abundance of the top 20 genera between rats pre-and post-antibiotics (Figure 3B). Consistent with the heatmap findings, the genera showing the most significant differences in relative abundance (LDA > 3.5, *p* < 0.05) included *Lactobacillus*, *Muribaculaceae*, and *Romboutsia*, which decreased following antibiotic treatment, and *Escherichia–Shigella* and *Enterococcus*, which increased (Figure 3B). Several of these genera were also identified in genus-level co-occurrence networks, revealing patterns of positive associations among taxa in control (Figure S2A) and antibiotic-treated (Figure S2B) rats.

**Figure 4:**
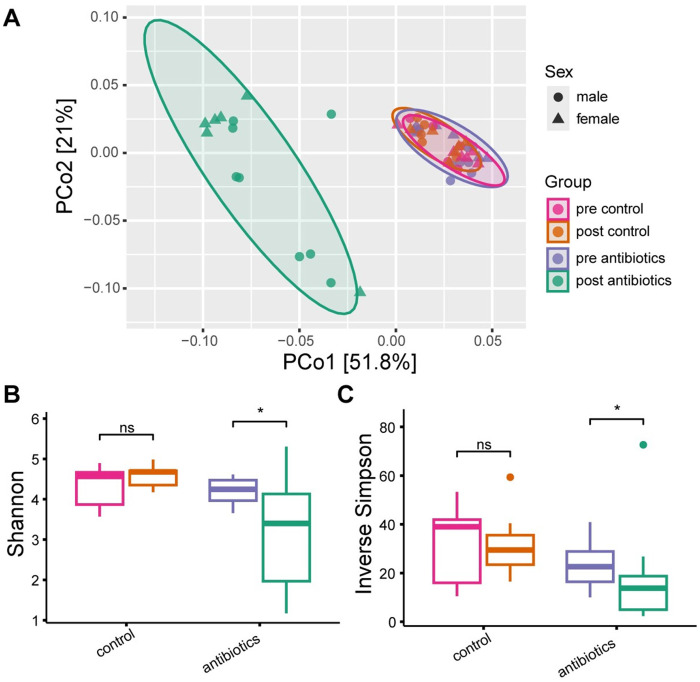
Antibiotic treatment alters the community structure of the rat fecal microbiome. **A)** Principal Coordinate Analysis (PCoA) of fecal microbiota from rats pre-treatment or post-water or antibiotic treatment, based on weighted UniFrac distances of Amplicon Sequence Variant (ASV) abundances (PERMANOVA, *p* < 0.001). **B)** Shannon diversity index and **C)** Inverse Simpson diversity index calculated from rat fecal samples. Statistical significance between groups was assessed using the Wilcoxon rank-sum test (*p < 0.01).

As shown by the Principal Coordinate Analysis (PCoA) plot, a significant shift in the structure of the fecal microbiome occurred due to antibiotic treatment (PERMANOVA, *p* < 0.01; Figure 4A). Pre-control and pre-antibiotics samples compared to post-antibiotics revealed a statistically significant difference (*p* = 0.001), pre-control samples compared to post-control did not reveal a statistically significant difference (*p* = 0.63; Figure 4A). Additionally, there was a significant decrease in microbiome diversity after antibiotic treatment (Wilcoxon rank-sum test, *p* < 0.01) as addressed by Shannon (Figure 4B) and Inverse Simpson (Figure 4C) diversity metrics. Among post-antibiotic treated rats, there was no statistically significant difference in microbiome structure between males and females as shown by the PCoA plot (Figure S3A). For the Shannon diversity index (Figure S3B), both males and females revealed a significant decrease for post-antibiotics samples compared to post-control. Females also displayed a significant decrease for pre-antibiotics compared to post-control. For the Inverse Simpson diversity index (Figure S3C), no statistically significant difference was determined for males. Females had a decreased Inverse Simpson diversity index for post-antibiotics compared to post-control.

Short chain fatty acid (SCFA) analysis in serum as well as fecal samples further revealed the effects of antibiotic treatment on the gut microbiome. All SCFAs were decreased following antibiotic treatment in both sexes. For example, the mean fecal acetic acid level for the Male Antibiotics group was 53.83 nmol/mg pre-treatment and 6.37 nmol/mg post-treatment. The mean fecal acetic acid level for the Female Antibiotics group was 59.01 nmol/mg pre-treatment and 0.99 nmol/mg post-treatment. Table 2 displays those differences in fecal SCFAs (# indicates a trend, *p*-values are reported); the top of the table includes mean values for fecal SCFAs (nmol/mg) by group, from samples collected prior to the antibiotic or control (water) administration. Below, are the mean fecal SCFA values (nmol/mg) by group, post-treatment, followed by statistical comparisons for the post-treatment values. No significant differences were determined for pre-treatment values. Finally, the bottom of the table includes pre vs. post-treatment statistical comparisons. Table 3 displays serum SCFA values (μM) and statistical comparisons. Serum was only collected at the end of the study. Therefore, we do not have pre vs. post-treatment comparisons, only serum SCFA comparisons by group. Three serum SCFA levels were below detection for antibiotics-treated groups: propionic acid, butyric acid, and valeric acid. Interestingly, some serum SCFAs were not significantly changed by antibiotic treatment. For example, iso-butyric acid levels were slightly increased (not statistically significant) for antibiotics-treated groups compared to control (water) groups, in both sexes. Importantly, we also observed a sex difference in serum iso-caproic acid in water groups as well as a sex difference in serum octanoic acid in antibiotics groups. Females had higher levels of iso-caproic acid compared to males, whereas males had higher levels of octanoic acid compared to females.

Serum bile acid levels (μM) were also analyzed, many were reduced by antibiotic treatment, particularly for male rats (Table 4). For example, the mean serum cholic acid level for Male Control was 11.41 μM vs. 0.037 μM for Male Antibiotics. The mean serum cholic acid level for Female Control was 5.48 μM vs. 0.023 μM for Female Antibiotics. There were a wide range of serum cholic acid values for Male Control and particularly, Female Control. Therefore, a statistically significant difference for Female Control vs. Female Antibiotics was not determined. Male Antibiotics had significantly lower serum cholic acid levels compared to Male Control (*p* = 0.0360). Raw values for all of these measurements are posted on FigShare: 10.6084/m9.figshare.30306424. Characterization of fecal SCFAs, serum SCFAs, and serum bile acids between males and females, with and without antibiotics, provides important information and characterization related to the gut microbiome and metabolism.

**Figure 5:**
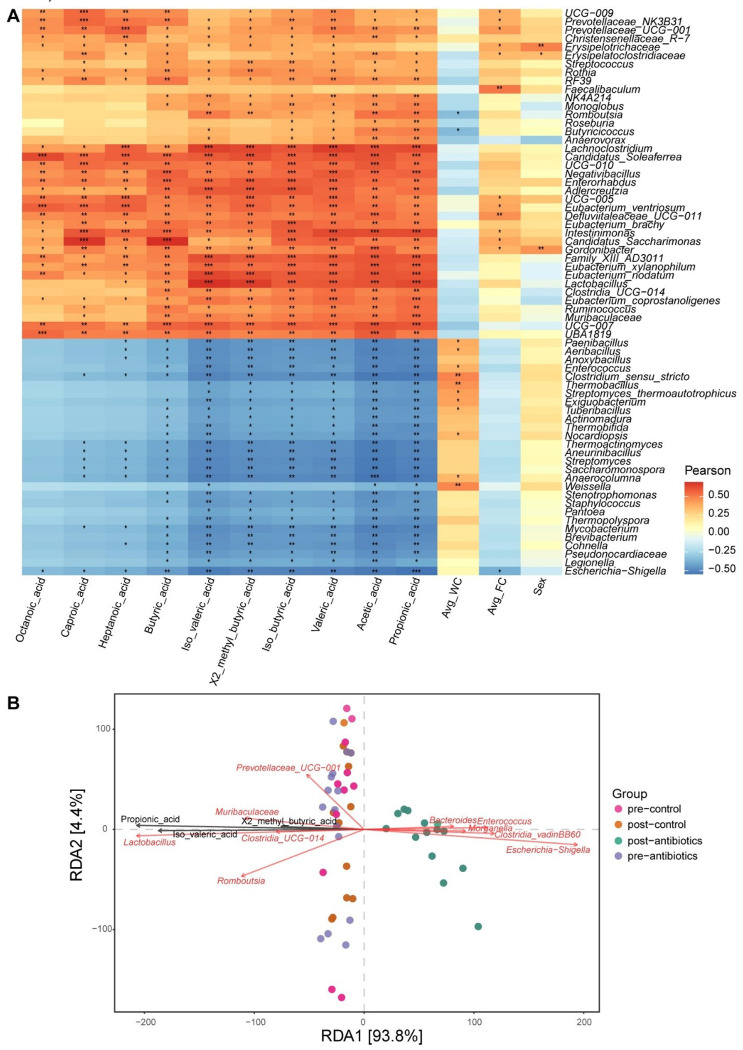
Impact of hedonic feeding and related variables on the gut microbiome. **A)** Correlation analysis between SCFA content, food and water consumption, sex, and microbial genera. Pearson correlation coefficients were calculated, and only taxa with *p* < 0.001 are shown. **B)** Redundancy analysis (RDA) plot illustrating the relationships between microbial community structure and three SCFAs identified as significant drivers.

Correlation analysis revealed significant associations between SCFA content, water, and home cage food consumption, and numerous specific microbial taxa (Figure 5A), whereas only a few genera were correlated with sex. SCFA content was positively correlated with 39 different genera, including the most abundant ones, such as *Lactobacillus, Muribaculaceae, Romboutsia, Clostridia UCG-014, Bacteroides, and Ruminococcus*. Consistent with previous results, fecal SCFA and its positively associated taxa decreased after the antibiotic treatment. Thus, SCFA content showed an inverse correlation with the genera that increase their abundances in the post-antibiotic conditions (i.e.: *Clostridium sensu stricto, Escherichia-Shigella, Enterococcus, Staphylococcus*, etc.).

To further investigate the influence of hedonic feeding and related variables on microbial composition, a redundancy analysis (RDA) was performed (Figure 5B). The model identified propionic, isovaleric, and 2-methylbutyric acids as the most significant drivers of the community structure across treatments (adj. *p*-value < 0.01). These 3 fatty acids were the main predictors of the microbial communities for pre-control, pre-antibiotics, and post-control samples, while they showed a negative association with post-antibiotic samples.

## Discussion

First, we determined that females have a higher demand at null cost for the high fat palatable reward compared to males. We have previously shown this sex difference [8]. However, in our previous study, males and females were mildly food restricted in order to encourage lever pressing for the palatable rewards. Here, we provided ad libitum access to homecage food and continued to see lever pressing behavior to produce full demand curves. However, more males (n = 11) failed to reach criteria during BE training with ad libitum homecage feeding compared to females (n = 2) due to lower lever-pressing for the pellet, further supporting the finding that females have a higher demand for this palatable reward compared to males. Second, we characterized hedonic feeding, homecage feeding, fecal microbiome composition and diversity, serum and fecal SCFA composition, and serum bile acid composition following disruption of the gut microbiome with antibiotics. An antibiotic cocktail was utilized to disrupt the gut microbiome and test whether diversity of the gut microbiome impacted hedonic feeding behavior. In order to control for time between BE sessions as well as repeated sessions, we also included a subgroup of animals given normal drinking water for one month instead of the antibiotic cocktail. We did not determine a significant effect of antibiotics on hedonic feeding, despite disruption to the fecal microbiome. A mixed-effects analysis for time x sex x treatment and Tukey’s multiple comparisons test was applied; Female Control and Female Antibiotics had a significantly higher Q_0_ value compared to Male Control pre-treatment. No significant differences were determined between Female Control and Female Antibiotics, Male Control and Male Antibiotics, or Male Antibiotics and Female Antibiotics pre-treatment. No statistically significant differences in Q_0_ were determined for any group post-treatment. We did not determine any statistically significant differences in α between groups or between time points. Male Antibiotics had a slightly higher mean α value after antibiotics treatment, indicative of lower motivation, but that is not statistically significant. Importantly, this behavior analysis focused on hedonic feeding behavior; animals were not obese.

Females consumed more water (normalized to their weight) during antibiotic treatment. Therefore, females received a higher dose of antibiotics compared to males. We noted temporary, reduced water consumption in male rats as well as weight loss, which may be related to an aversion to the taste of antibiotics. Previous work has shown that changes in drinking and feeding are not fully attributable to the bitter taste of antibiotics [42]. Studies have shown that females exhibit enhanced taste perception compared to males, particularly for bitter flavors, as well as sweet and salty tastes [43, 44].

Complementing enhanced taste perception for females, enhanced olfaction has also been shown for females [45–47]. Therefore, decreased consumption of the antibiotic cocktail is likely not fully explained by taste aversion in males. In a study by Parodi et al. (2022), male and female Sprague-Dawley rats had significantly different responses to eight days of an antibiotic cocktail in their drinking water. Their antibiotic cocktail included the same antibiotics as our cocktail, however, it also included 1 g/L metronidazole. We chose not to include metronidazole in our current study given that it crosses the blood-brain barrier [48]. Similar to our findings, males and females exhibited significant differences in microbiome composition as well as body weight in response to the antibiotics. Their study also determined that males and females experienced weight loss during antibiotic treatment, however, females recovered their body weights after antibiotic treatment ended, while males did not [38].

We collected fecal samples during both rounds of BE testing. We compared diversity and composition of the fecal microbiome between groups and time points. We, and others, have previously observed baseline sex differences in fecal microbiome composition and diversity for C57Bl/6 mice [36, 49–52]. We did not observe striking baseline sex differences in our current study with Sprague-Dawley rats. We hypothesized that sex differences in the gut microbiome contribute to sex differences in hedonic feeding. Given that there were no baseline sex differences in the fecal microbiome composition and disruption of the gut microbiome with antibiotics did not result in significant differences in hedonic feeding, our hypothesis was not supported. It may still be possible that the gut microbiome contributes to sex differences in hedonic feeding, even though our results do not support this claim. We consider a number of possibilities and limitations of our current study: 1) fecal samples were collected during BE testing in order to make within-animal comparisons (for example, pre-antibiotics and post-antibiotics within the same subject). A future study could, instead, collect samples directly from the colon to more accurately characterize aerobic and anaerobic bacteria. However, in this alternative experimental design, we could only evaluate behavior one time and then euthanize the animal to collect the samples. Still, there are other bacteria that are not fully captured with our current method. Furthermore, the gut microbiome is influenced by viruses, yeast, fungi, and archaea; there could be sex differences in these microorganisms that impact hedonic feeding. 2) Our current study evaluated adult rats and does not take into account the timeline during which the microbiome affects the circuitry for hedonic feeding. If the microbiome shapes hedonic feeding circuitry, it is likely that this occurs early in development. The behavioral economics paradigm was implemented during adulthood due to the complexity of the task and size of the operant chamber/position of levers that could be more difficult for younger rats to complete. The hedonic feeding circuitry could be less plastic during adulthood. 3) The antibiotic cocktail was administered in the drinking water. An alternative experimental design could include oral gavage of the antibiotics to control dosage. However, repeated oral gavage can be stressful and can affect the gastrointestinal tract. Therefore, we chose the less invasive strategy of antibiotic cocktail administration via their homecage water bottles. We did observe decreased consumption by male rats compared to female rats when we normalized consumption and dosage to body weight. Ultimately, we observed disruption of the fecal microbiome for both male and female rats. We also observed decreased SCFAs in male and female antibiotic-treated rats, indicating disruption of the gut microbiome to impact these metabolites in both sexes.

In addition to analyzing fecal microbiome diversity and genus-level composition, we characterized fecal SCFAs, serum SCFAs, and serum bile acids. SCFA analysis can provide more information about microbiome metabolism. For example, it is known that *Clostridium, Butyrivibrio*, and *Eubacterium* are major producers of butyric acid [53]. Following antibiotic administration, we determined significant decreases in fecal and serum SCFAs for both males and females. We did determine a significant sex difference for serum iso-caproic acid for control groups and serum octanoic acid for antibiotic groups. Females had higher levels of iso-caproic acid compared to males, and higher levels following antibiotic treatment. Males had higher levels of octanoic acid compared to females, and higher levels following antibiotic treatment. We did not observe sex differences for these SCFAs in fecal samples. Fecal samples had significantly lower iso-caproic levels and octanoic acid levels for both sexes after antibiotics. However, in a previous study with C57Bl/6 mice we did observe a sex x dietary treatment/antibiotic treatment effect for fecal octanoic acid (among other SCFAs): males fed a low-fat diet had higher levels of octanoic acid than all other groups [35]. The observed differences in serum SCFAs in the current study could be due to differences in fatty acid metabolism, which has already been shown to be different in males and females [35, 54, 55]. Fatty acid utilization, lipid sensing, and lipid taste can impact hedonic feeding [56–58].

We further analyzed serum bile acid concentrations. Bile acids are synthesized in the liver, affect gastrointestinal hormone secretion, and they are also known to impact appetite, glucose metabolism, and lipid metabolism. For example, supplementation with cholic acid has been shown to reverse adiposity in mice administered a high fat diet [59]. In that study, chow-fed animals were not affected by the cholic acid supplementation. The high fat-fed, obese mice exhibited decreased white adipose tissue as well as improved glucose tolerance after cholic acid supplementation [59]. On the other hand, administration of deoxycholic acid to high fat-fed mice revealed increased hepatic ER stress, reduced hepatic insulin signaling, and impaired glucose homeostasis [60]. Bile acid subtypes have variable effects on glucose metabolism. Here, we determined significant changes to many bile acid subtypes following antibiotic administration, particularly for male rats. Antibiotics had less of an effect on bile acid concentrations for female rats. For example, cholic acid was significantly reduced in antibiotic-treated males (*p* = 0.0360), but not in antibiotic-treated females (*p* = 0.7208) because females already had lower levels of cholic acid. We also determined a significant sex difference: Female Control had higher levels of lithocholic acid compared to Male Control (*p* = 0.0007). Lithocholic acid (LCA) was significantly reduced post-antibiotics for female rats (*p* = 0.0020). Interestingly, LCA is increased after calorie restriction, it has been shown to activate AMPK [61], it decreases blood glucose levels, and it increases plasma GLP-1 levels [62]. Furthermore, it is known that *Lactobacillus, Clostridium* and *Eubacterium* species convert cholic acid and chenodeoxycholic acid to lithocholic acid [63]. We also determined significant sex differences in taurochenodeoxycholic (TCDCA). Females had higher levels of TCDCA compared to males, which increased even further with administration of antibiotics; that increase in TCDCA post-antibiotics was not observed for males. TCDCA is a conjugated bile acid that has been shown to increase following high fat feeding [64]. Previous work has demonstrated that microbiome changes due to high fat feeding can lead to increased TCDCA levels which can activate the farnesoid X receptor (FXR) and induce insulin resistance [64, 65]. The composition of the gut microbiome has been shown to impact feeding behaviors. We did not determine major sex differences in the gut microbiome here that could contribute to our observed sex differences in hedonic feeding.

## Conclusions

We determined that female Sprague-Dawley rats have a higher demand at null cost for a high fat palatable reward compared to male Sprague-Dawley rats. We also determined that administration of antibiotics did not significantly change hedonic feeding behavior; however, sex differences in Q_0_ values did not persist following antibiotic administration. Furthermore, we did not observe striking baseline sex differences for gut microbiome composition and diversity in our current study with Sprague-Dawley rats. This brings to question whether the gut microbiome contributes to sex differences in hedonic feeding. More research will be necessary for network factors such as microbiome – bile acid effects on feeding that exhibit sex differences.

## Supplementary Material

This is a list of supplementary files associated with this preprint. Click to download.

• FigureS1S3.docx

• Table14.docx

## Figures and Tables

**Table 1.** Average Number of Active/Inactive Lever Responses and Pellets Earned During 1-Hour FR Training Sessions Prior to BE Testing. Values are n (SEM).

**Table 2.** Fecal SCFA Composition Analyses. Mean fecal SCFA levels reported in nmol/mg. Pre-treatment: Male Control (n = 3), Male Antibiotics (n = 8), Female Control (n = 6), Female Antibiotics (n = 6). Posttreatment: Male Control (n = 5), Male Antibiotics (n = 8), Female Control (n = 5), Female Antibiotics (n = 7).

**Table 3.** Serum SCFA Composition Analyses. Mean serum SCFA levels reported in µM. Male Control (n = 6), Male Antibiotics (n = 7), Female Control (n = 7), Female Antibiotics (n = 5).

**Table 4.** Serum Bile Acid Composition Analyses. Mean serum bile acid levels reported in µM. Male Control (n = 6), Male Antibiotics (n = 7), Female Control (n = 6), Female Antibiotics (n = 5).

## Data Availability

Raw sequence data have been deposited in the Sequence Read Archive (Project PRJNA1337140): https://www.ncbi.nlm.nih.gov/sra?linkname=bioproject_sra_all&from_uid=1337140. The behavior, short chain fatty acid, and bile acid datasets are available at FigShare: 10.6084/m9.figshare.30306424.
